# mTORC1-mediated downregulation of COX2 restrains tumor growth caused by TSC2 deficiency

**DOI:** 10.18632/oncotarget.8633

**Published:** 2016-04-07

**Authors:** Hongwu Li, Fuquan Jin, Keguo Jiang, Shuang Ji, Li Wang, Zhaofei Ni, Xianguo Chen, Zhongdong Hu, Hongbing Zhang, Yehai Liu, Yide Qin, Xiaojun Zha

**Affiliations:** ^1^ Department of Otorhinolaryngology, Head & Neck Surgery, The First Affiliated Hospital of Anhui Medical University, Hefei, China; ^2^ Department of Biochemistry & Molecular Biology, School of Basic Medicine, Anhui Medical University, Hefei, China; ^3^ Department of Otorhinolaryngology, Head & Neck Surgery, The Fourth Affiliated Hospital of Anhui Medical University, Hefei, China; ^4^ School of Pharmacy, Anhui Medical University, Hefei, China; ^5^ Department of Nephrology, The Third Affiliated Hospital of Anhui Medical University, Hefei, China; ^6^ Department of Urology, The First Affiliated Hospital of Anhui Medical University, Hefei, China; ^7^ Modern Research Center for Traditional Chinese Medicine, Beijing University of Chinese Medicine, Beijing, China; ^8^ State Key Laboratory of Medical Molecular Biology, Department of Physiology & Pathophysiology, Institute of Basic Medical Sciences, Chinese Academy of Medical Sciences & Peking Union Medical College, Beijing, China; ^9^ State Key Laboratory Incubation Base of Dermatology, Ministry of National Science and Technology, Hefei, China

**Keywords:** mTOR, STAT3, COX2, TSC, IL-6

## Abstract

Tuberous sclerosis complex (TSC), caused by loss-of-function mutations in the TSC1 or TSC2 gene, is characterized by benign tumor formation in multiple organs. Hyperactivation of mammalian target of rapamycin complex 1 (mTORC1) is the primary alteration underlying TSC tumors. By analyzing Tsc2-null mouse embryonic fibroblasts (MEFs) and rat uterine leiomyoma-derived Tsc2-null ELT3 cells, we detected evidence for the involvement of cyclooxygenase 2 (COX2) as a downstream target of mTORC1 in the development of TSC tumors. We showed that loss of TSC2 led to decreased COX2 expression through activation of an mTORC1/signal transducer and activator of transcription 3 (STAT3) signaling pathway. Overexpression of COX2 promoted proliferation and tumoral growth of Tsc2-null cells. COX2 knockdown inhibited the proliferation of the control cells. COX2 enhanced Tsc2-null cell growth through upregulation of interleukin-6 (IL-6). In addition, rapamycin in combination with celecoxib, a COX2 inhibitor, strongly inhibited Tsc2-deficient cell growth. We conclude that downregulation of COX2 exerts a protective effect against hyperactivated mTORC1-mediated tumorigenesis caused by the loss of TSC2, and the combination of rapamycin and celecoxib may be an effective new approach to treating TSC.

## INTRODUCTION

Tuberous sclerosis complex (TSC) is an autosomal dominant disease characterized by formation of benign tumors in multiple organs, including kidney, brain, and skin [1–3]. This disorder is caused by inactivating mutations in either of two tumor suppressor genes: *TSC1* or *TSC2* [4]. TSC1 and TSC2 protein form a functional complex that negatively regulates a small GTPase, Ras homologue enriched in brain (Rheb), through the GTPase-activating (GAP) activity of TSC2 [4, 5]. Disruption of the TSC1/TSC2 complex by inactivating mutations in either *TSC1* or *TSC2* leads to the accumulation of GTP-bound Rheb, which in turn activates mammalian target of rapamycin complex 1 (mTORC1) [6]. Hyperactivated mTORC1 signaling leads to uncontrolled cell growth and tumorigenesis, and it is therefore considered to be responsible for the tumor development in TSC [7, 8]. It is noteworthy that TSC patients rarely develop malignant lesions [9]. Although it is believed that the negative feedback inhibition of AKT by the dysregulated mTORC1 is the major reason for the benign nature of TSC tumors [9, 10], whether additional signaling molecules contribute to restrict tumor development remains less clear.

Cyclooxygenases (COXs) are a family of myeloperoxidases that catalyze the biosynthesis of prostaglandins (PGs) from arachidonic acid [11, 12]. So far, three COX isoforms have been identified. COX1 is constitutively expressed in a wide range of tissues and is responsible for maintaining basal PG levels for tissue homeostasis [11]. COX2 is an inducible isoform that produces PGs in inflammatory and tumorigenic settings [13]. COX3 is a splice variant of COX1 that encodes a truncated protein lacking enzymatic activity [12]. Among the COX family, COX2 has been shown to play a crucial role in carcinogenesis by promoting growth, survival, and metastasis of tumor cells [12]. Overexpression of COX2 has been reported in many tumor types [14–18]. Our aim in the present study was to determine the precise function of COX2 in TSC tumors. Our findings suggest downregulation of COX2 limits the development of TSC tumors, and a combination of rapamycin and celecoxib may be exploited as a novel regimen for the treatment of TSC.

## RESULTS

### TSC2 is a positive regulator of COX2

**Figure 1:**
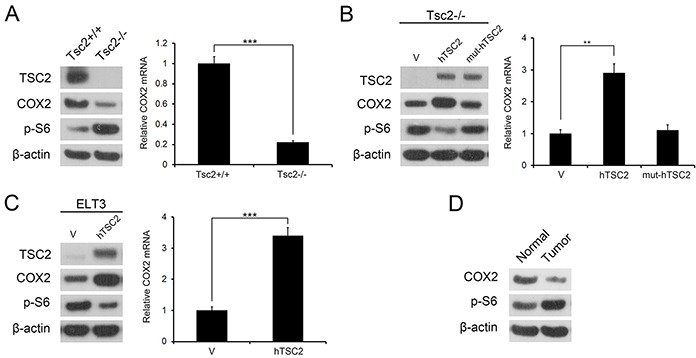
TSC2 positively regulates COX2 expression **A.** Tsc2+/+ and Tsc2−/− MEFs. **B.** pLXIN (V), pLXIN-hTSC2, or pLXIN-mut-hTSC2 retroviruses infected Tsc2−/− MEFs. **C.** ELT3 cells were transduced using retroviruses harboring hTSC2 in pLXIN or its control vector pLXIN (V). **A-C.** Cell lysates were subjected to immunoblotting with the indicated antibodies (left panel). COX2 mRNA was analyzed by qRT-PCR (right panel). Error bars indicate mean ± SD of triplicate samples. **P<0.01; ***P<0.001. **D.** Kidney cystadenomas (Tumor) and the adjacent normal tissues (Normal) from Tsc2+/− mice were immunoblotted with the indicated antibodies.

The fact that COX2 is frequently deregulated in tumors and that TSC is characterized by benign tumor formation in multiple organs prompted us to investigate the role of COX2 in the development of TSC tumors. We first checked the level of COX2 in Tsc2−/− MEFs and their control cells (Tsc2+/+ MEFs). As expected, immunoblotting analysis demonstrated that loss of TSC2 led to activation of mTORC1 signaling (p-S6 is an indicator of mTORC1 activity) (Figure 1A left panel). In addition, COX2 expression in Tsc2−/− MEFs was significantly lower than in the control cells (Figure 1A left panel). Moreover, qRT-PCR analysis showed that the downregulation of COX2 occurred at the transcriptional level (Figure 1A right panel). Ectopic expression of wild-type human TSC2 (hTSC2) normalized the p-S6 level and robustly restored expression of COX2 in Tsc2−/− MEFs (Figure 1B). By contrast, reintroduction of a patient-derived GAP domain mutant (N1651S) TSC2 (mut-hTSC2) had no effect on p-S6 levels or COX2 expression (Figure 1B). Consistent with those observations, ectopic expression of hTSC2 increased levels of both COX2 mRNA and protein in rat uterine leiomyoma-derived Tsc2-null ELT3 cells (Figure 1C). The relationship between TSC2 and COX2 was further verified *in vivo* by assessing the levels of COX2 in renal tumors and adjacent normal renal tissues from Tsc2+/− mice. As shown in Figure 1D, COX2 levels were lower within the tumors than in the adjacent normal tissues. Taken together, these data indicate that TSC2 positively regulates COX2.

### Loss of TSC2 downregulates COX2 through activation of mTORC1

**Figure 2:**
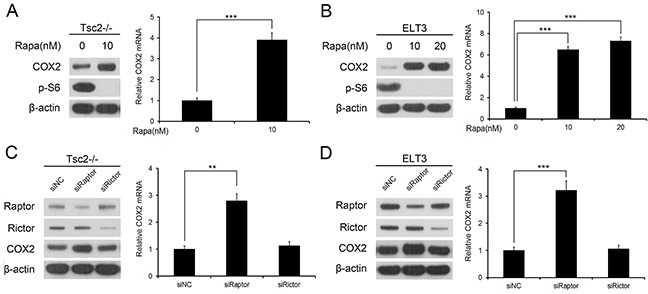
mTORC1 negatively regulates COX2 expression **A** and **B.** Tsc2−/− MEFs (A) or ELT3 cells (B) were treated for 24 h with or without the indicated concentration of rapamycin (Rapa). **C** and **D.** Tsc2−/− MEFs (C) or ELT3 cells (D) were transfected for 48 h with siRNA targeting Raptor or Rictor, or with the control siRNA (siNC). **A-D.** Cell lysates were subjected to immunoblotting with the indicated antibodies (left panel). COX2 mRNA was detected by qRT-PCR (right panel). Error bars indicate mean ± SD of triplicate samples. **P<0.01; ***P<0.001.

Because loss of TSC2 induced both hyperactivation of mTORC1 and downregulated of COX2, we speculated that mTORC1 negatively regulates the expression of COX2. To test this hypothesis, we first evaluated the effect of rapamycin, a specific mTORC1 inhibitor, on COX2 expression. As shown in Figure 2A, treating Tsc2−/− MEFs with rapamycin led to a marked reduction in mTORC1 activity and a concomitant increase in COX2 expression. Similarly, rapamycin dramatically upregulated COX2 expression in ELT3 cells (Figure 2B).

mTOR exists in two multiprotein complexes, rapamycin-sensitive mTORC1 and rapamycin-insensitive mTORC2 [19–21]. To further verify that it is indeed mTORC1 that mediates the negative regulation of COX2 downstream of TSC2, we examined COX2 levels in Raptor- (a specific component of mTORC1) and Rictor (a specific component of mTORC2)-knockdown Tsc2−/− MEFs. As shown in Figure 2C, transfecting Tsc2−/− MEFs with small interfering RNAs (siRNAs) targeting Raptor increased COX2 levels within the cells, whereas Rictor siRNAs had little effect on COX2 expression. Similarly, knocking down Raptor, but not Rictor, led to upregulated expression of COX2 in ELT3 cells (Figure 2D). It thus appears that hyperactivated mTORC1 signaling is responsible for the downregulation of COX2 in Tsc2-null cells.

### mTORC1 downregulates COX2 through activation of STAT3

**Figure 3:**
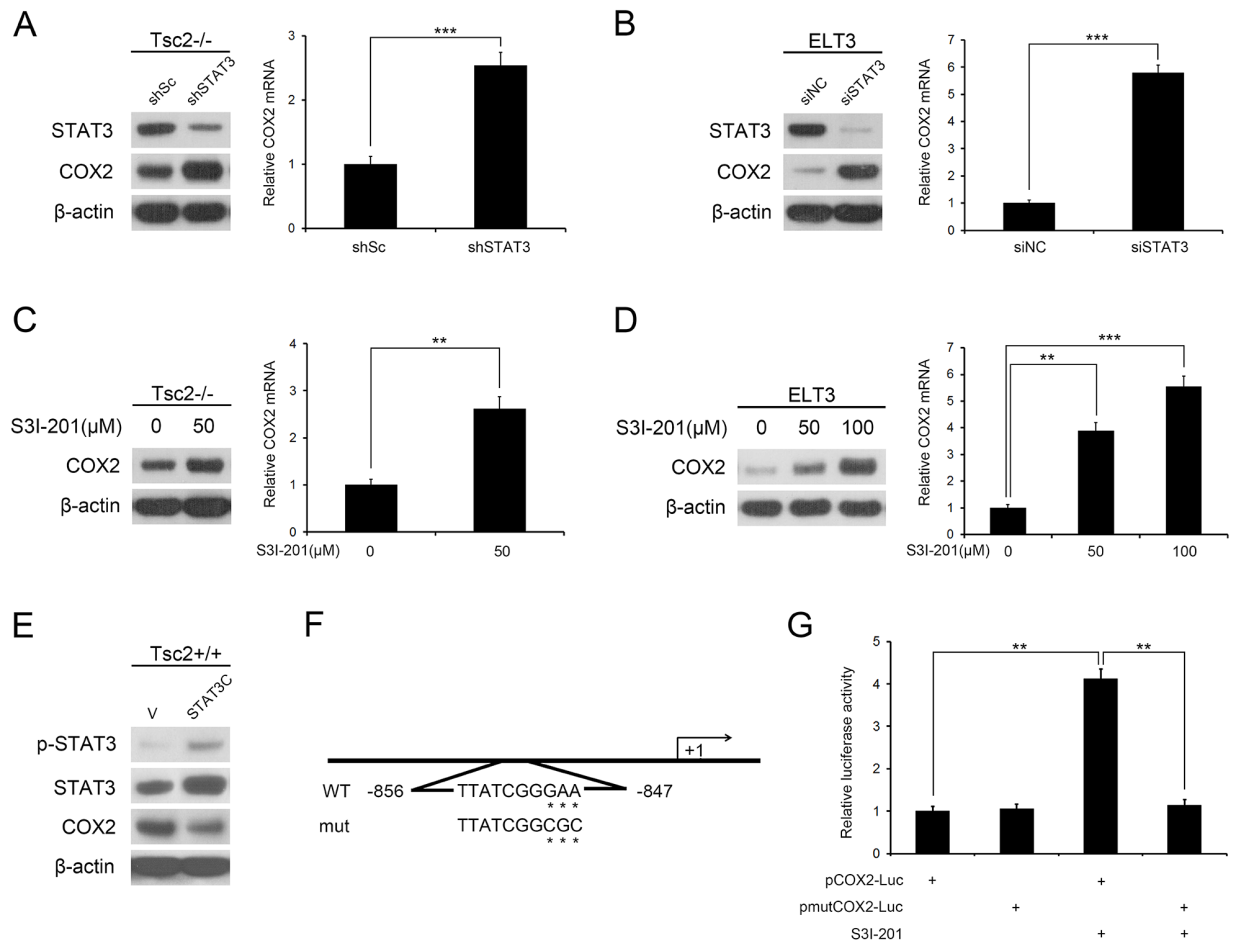
mTORC1 downregulates COX2 expression through activation of STAT3 **A.** Tsc2−/− MEFs were infected with lentiviruses harboring vectors encoding STAT3 shRNA (shSTAT3) or the control shRNA (shSc). **B.** ELT3 cells were transfected for 48 h with STAT3 siRNA or the control siRNA (siNC). **C** and **D.** Tsc2−/− MEFs (C) or ELT3 cells (D) were treated for 24 h with or without the indicated concentration of S3I-201. **A-D.** Cell lysates were subjected to western blot analysis using the indicated antibodies (left panel). COX2 mRNA was detected by qRT-PCR (right panel). **E.** Tsc2+/+ MEFs were infected with retroviruses harboring pBabe-puro encoding a constitutively activated STAT3 (STAT3C) or its control vector pBabe-puro (V). The proteins were detected by immunoblotting with the indicated antibodies. **F.** Schematic representation of the putative wild-type (WT) and mutated (mut) STAT3-binding sites in the promoter of rat COX2 gene. **G.** ELT3 cells were pretreated for 24 h with or without 50 μM S3I-201 and then cotransfected with 200 ng of pCOX2-Luc or pmutCOX2-Luc reporter plasmid and 20 ng of pRL-TK plasmid. Relative luciferase activity was evaluated 24 h after transfection. Error bars indicate mean ± SD of triplicate samples. **P<0.01; ***P<0.001.

We previously reported that loss of TSC2 stimulated STAT3 activity through activation of mTORC1 signaling [22, 23]. To identify downstream targets of the TSC2/mTORC1/STAT3 signaling pathway, we used lentiviral vectors encoding siRNAs targeting STAT3 in Tsc2−/− MEFs. Subsequent gene expression profiles showed that COX2 levels were higher in the STAT3-knockdown cells than in control cells (Supplementary Table S1). Western blot and qRT-PCR analyses confirmed that depletion of STAT3 dramatically increased expression of COX2 in Tsc2−/− MEFs (Figure 3A). In similar fashion, knocking down STAT3 in ELT3 cells markedly reduced STAT3 and increased of COX2 (Figure 3B). Moreover, inhibition of STAT3 activity using S3I-201, a specific STAT3 inhibitor, also dramatically increased COX2 expression in both Tsc2−/− MEFs and ELT3 cells (Figure 3C and 3D). Conversely, ectopic expression constitutively activated STAT3 (STAT3C) [24] decreased expression of COX2 in Tsc2+/+ MEFs (Figure 3E).

To further elucidate the mechanisms involved in the regulation of COX2 by STAT3, we analyzed the 5′-flanking sequence of the COX2 gene upstream of the start transcription site. A conserved STAT3 binding sequence (TTATCGGGAA; −856/−847) was detected within the promoter of the rat COX2 gene (Figure 3F). We then cloned the rat COX2 promoter (from -994 to +121 bp) into a luciferase reporter vector and evaluated the effect of STAT3 on promoter activity. Inhibition of STAT3 by S3I-201 increased luciferase activity (Figure 3G), but the enhanced transcriptional activity was attenuated when the putative STAT3-binding sequence was mutated (Figure 3G). These results demonstrate that STAT3 downregulates COX2 expression downstream of TSC2/mTORC1 in Tsc2-null cells.

### Downregulation of COX2 inhibits the proliferation, colony formation, and tumor growth of Tsc2-deficient cells

**Figure 4:**
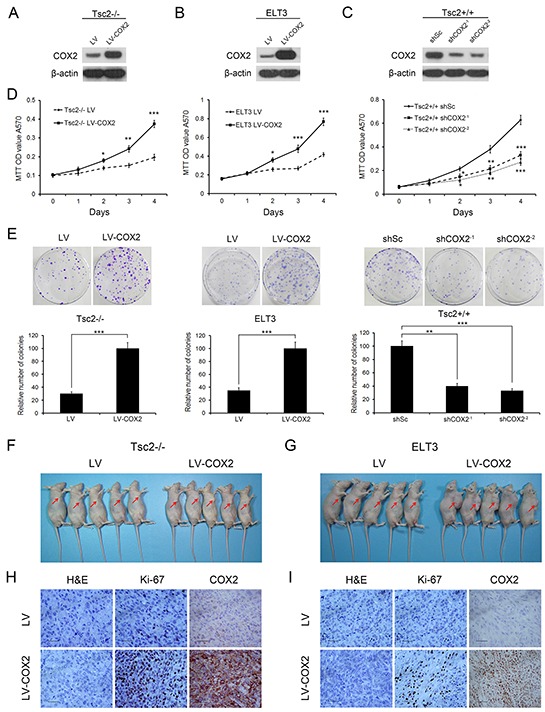
Decreased COX2 inhibits the cell proliferation, colony formation, and *in vivo* tumorigenicity of Tsc2-deficient cells **A** and **B.** Tsc2−/− MEFs (A) or ELT3 cells (B) were infected with lentivirus harboring a vector encoding COX2 (LV-COX2) or the empty vector (LV). **C.** Two independent shRNAs targeting COX2 (shCOX2^−1^ and shCOX2^−2^) or a control shRNA (shSc) were stably expressed in Tsc2+/+ MEFs. A-C. Cell lysates of the indicated cells were subjected to immunoblotting with the indicated antibodies. **D.** Proliferation of the indicated cells was examined using MTT assays. E. Representative images (upper panel) and quantifications (lower panel) of crystal violet-stained colonies formed by the indicated cells. Error bars indicate mean ± SD of triplicate samples. *P<0.05; **P<0.01; ***P<0.001. **F** and **G.** COX2-overexpressing Tsc2-null MEFs (F) or ELT3 cells (G) and the corresponding control cells were inoculated subcutaneously into nude mice, which were then monitored for tumor growth. **H** and **I.** Formalin-fixed, paraffin-embedded Tsc2-null MEF (H) and ELT3 cell (I) tumor sections were subjected to H&E and immunohistochemical staining, as indicated. Representative images were presented. Scale bar, 50 μm.

To evaluate the potential role of COX2 in TSC tumors, Tsc2-null MEFs were transfected with lentiviral vectors for overexpressing COX2, which was confirmed by western blot (Figure 4A). As expected, the ectopic COX2 expression markedly increased cell proliferation, as demonstrated by MTT assays (Figure 4D left panel) and colony formation assay (Figure 4E left panel). Similar results were obtained with ELT3 cells overexpressing COX2 (Figure 4B, 4D middle panel, and 4E middle panel).

The positive effect of COX2 on cell proliferation was confirmed using two different shRNAs targeting COX2 (shCOX2^−1^ and shCOX2^−2^) in Tsc2+/+ MEFs. Western blot analysis revealed that both shCOX2^−1^ and shCOX2^−2^ dramatically suppressed expression of COX2 (Figure 4C), while MTT and colony formation assays showed that the COX2 depletion led to a decrease in cell proliferation (Figure 4D right panel and 4E right panel). Inhibition of COX2 activity using celecoxib also significantly suppressed the proliferation of Tsc2+/+ MEFs (Supplementary Figure S1).

To evaluate the *in vivo* effects of COX2 on the tumoral growth of Tsc2-null cells, Tsc2−/− MEFs transfected with lentiviral vector encoding COX2 or empty vector were subcutaneously injected into the right anterior armpit of nude mice, and tumor growth was monitored. The results showed that the tumorigenic capacity of Tsc2−/− MEFs overexpressing COX2 was dramatically enhanced as compared to the control cells (Figure 4F and Supplementary Figure S2). Immunohistochemical analysis revealed that tumor tissues formed by injection of COX2-overexpressing Tsc2−/− MEFs exhibited much stronger staining for Ki-67 than those formed by the control cells (Figure 4H). And similar results were obtained when ELT3 cells transfected with COX2- or control-lentivirus were inoculated into nude mice (Figure 4G, 4I and Supplementary Figure S2).

### IL-6 is a downstream target of COX2 in Tsc2-null cells

**Figure 5:**
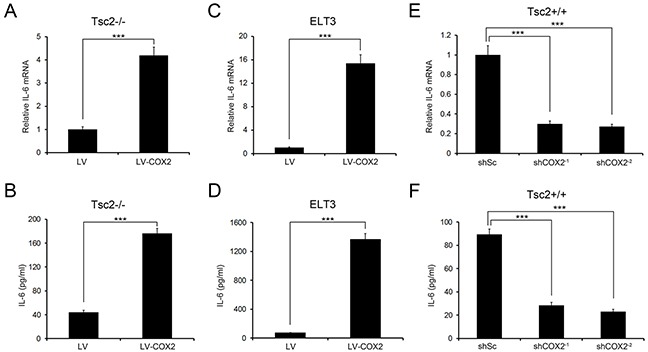
IL-6 is a downstream target of COX2 **A-D.** Tsc2−/− MEFs (A and B) or ELT3 cells (C and D) were infected with lentiviruses harboring pLVX-IRES-Puro encoding COX2 (LV-COX2) or its control vector pLVX-IRES-Puro (LV). **E** and **F.** Tsc2+/+ MEFs were infected with lentiviruses harboring vectors encoding non-targeting control shRNA (shSc) or shRNAs for knockdown of COX2 (shCOX2^−1^ and shCOX2^−2^). **A, C**, and **E.** IL-6 mRNA levels in the indicated cells were examined by qRT-PCR. **B, D**, and **F.** IL-6 levels in the cell supernatants of the indicated cells were detected using an ELISA. Error bars indicate mean ± SD of triplicate samples. ***P<0.001.

To identify molecules or signaling pathways involved in the increased growth capacity of COX2-overexpressing cells, we performed genome-wide expression analysis of Tsc2−/− MEFs and ELT3 cells overexpressing COX2 and the corresponding control cells. Of the 41 commonly upregulated genes, IL-6 was significantly increased in COX2-overexpressing cells as compared to the control cells (Supplementary Table S2). qRT-PCR analysis confirmed that ectopic expression of COX2 led to a marked upregulation of IL-6 in Tsc2-deficient cells (Figure 5A and 5C). Furthermore, using an ELISA we determined that IL-6 levels were higher in the cell culture supernatants of COX2-overexpressing Tsc2-null MEFs and ELT3 cells than in those of control cells (Figure 5B and 5D). Conversely, COX2 depletion led to reduced IL-6 expression and secretion in Tsc2+/+ MEFs (Figure 5E and 5F). These results suggest IL-6 is a downstream target of COX2 in Tsc2-deficient cells.

### Downregulation of COX2 reduces the proliferative capacity of Tsc2-null cells through inhibition of IL-6

**Figure 6:**
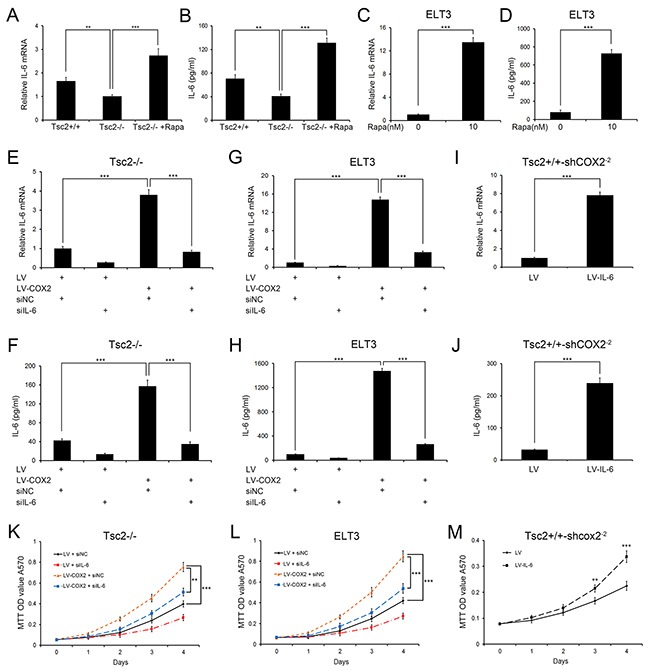
IL-6 mediates the effect of downregulating COX2 on Tsc2-null cell growth **A** and **B.** Tsc2+/+, Tsc2−/− and rapamycin (Rapa)-treated (10 nM for 24 h) Tsc2−/− MEFs. **C** and **D.** ELT3 cells were treated for 24 h with or without 10 nM rapamycin (Rapa). **E-H.** LV- or LV-COX2-expressing Tsc2−/− MEFs (E and F) or ELT3 cells (G and H) were transfected for 48 h with IL-6 siRNAs or control siRNA (siNC). **I** and **J.** Tsc2+/+-shCOX2^−2^ MEFs were infected with LV or LV-IL-6. **A, C, E, G**, and **I.** qRT-PCR analysis of IL-6 mRNA levels in the indicated cells. **B, D, F, H**, and **J.** Cell supernatants from the indicated cells were collected, and IL-6 levels were determined using an ELISA. **K-M.** Proliferation of the indicated cells was examined using MTT assays. Error bars indicate mean ± SD of triplicate samples. **P<0.01; ***P<0.001.

To elucidate the functional role of the COX2/IL-6 signaling pathway in the development of TSC tumors, we next assessed the level of IL-6 in Tsc2−/− MEFs and the control cells. As shown in Figure 6A and 6B, loss of TSC2 led to downregulation of IL-6 expression and secretion, and IL-6 levels were rescued through rapamycin treatment. Similar results were obtained with ELT3 cells (Figure 6C and 6D). We therefore speculated that reducing COX2/IL-6 signaling restrained proliferation of Tsc2-null cells.

We next used IL-6 siRNAs to knock down expression and secretion of IL-6 in COX2-overexpressing Tsc2−/− MEFs (Figure 6E and 6F). Depletion of IL-6 attenuated the accelerated cell proliferation driven by COX2 overexpression (Figure 6K). Again, similar results were obtained with ELT3 cells (Figure 6G, 6H and 6L). Furthermore, when we used a lentiviral vector to ectopically express IL-6 in Tsc2+/+-shCOX2^−2^ MEFs (Figure 6I and 6J), subsequent MTT assays demonstrated that the overexpression of IL-6 promoted cell proliferation (Figure 6M). These data indicate that downregulation of COX2 reduces the growth capacity of Tsc2-deficient cells at least partially through suppression of IL-6 expression.

### Rapamycin in combination with celecoxib strongly inhibits growth of Tsc2-deficient cells

**Figure 7:**
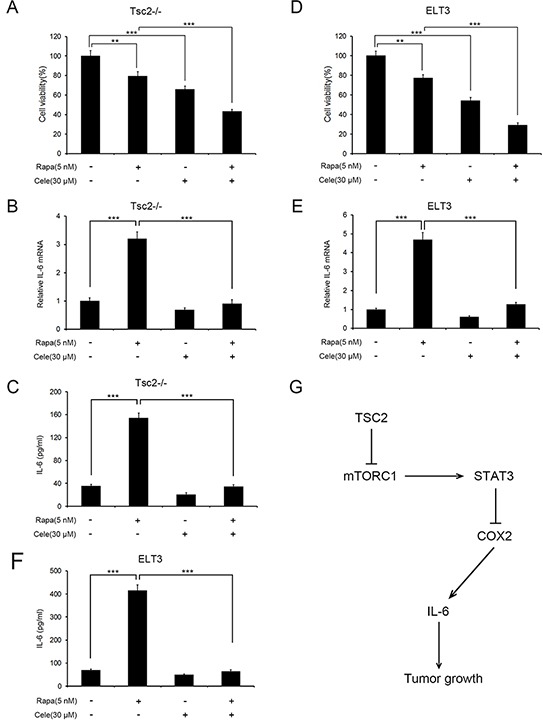
The combination of rapamycin and celecoxib more effectively suppresses growth of Tsc2-deficient cells than either substance alone **A-F.** Tsc2−/− MEFs (A-C) or ELT3 (D-F) cells were treated for 48 h with a combination of 5 nM rapamycin (Rapa) and 30 μM celecoxib (Cele) or either compound alone. A and D. Cell viability was assessed using MTT assays. **B** and **E.** IL-6 mRNA levels were determined using qRT-PCR. **C** and **F.** IL-6 levels in cell supernatants were determined using an ELISA. Error bars indicate mean ± SD of triplicate samples. **P<0.01; ***P<0.001. **G.** Schematic illustration of the TSC2/mTORC1/STAT3/COX2/IL-6 pathway regulated tumorigenesis.

Because rapamycin treatment markedly increased COX2 expression, we next examined whether the combination of rapamycin and celecoxib would achieve a better inhibition effect on the growth of Tsc2−/− MEFs. As shown in Figure 7A, the combination of 5 nM rapamycin and 30 μM celecoxib exerted a stronger inhibitory effect on the growth of Tsc2−/− MEFs than either compound alone. qRT-PCR and ELSIA analysis showed that celecoxib treatment abolished the upregulated expression and secretion of IL-6 triggered by rapamycin (Figure 7B and 7C). Rapamycin and celecoxib administered in combination similarly potentiated the cytotoxicity in ELT3 cells (Figure 7D). As in the MEFs, the increased IL-6 expression and secretion induced by rapamycin was attenuated by celecoxib administration in ELT3 cells (Figure 7E and 7F). Thus the combination of rapamycin and celecoxib inhibited the growth of Tsc2-deficient cells more effectively than either drug alone.

## DISCUSSION

Activation of mTORC1 caused by loss of TSC2 leads to significantly downregulated AKT activity, and restoration of AKT activity potentiates tumoral growth of Tsc2-deficient cells [9]. It is therefore thought that the reduced AKT activity resulting from mTORC1 activation delays tumor development in TSC [9, 10]. However, the extent to which other molecules in addition to AKT, downstream of mTORC1, contribute to restraining tumor development in TSC remained largely unknown. In the present study, we showed that loss of TSC2 led to downregulation of COX2 though activation of mTORC1 (Figure 2), whereas ectopic expression of COX2 enhanced the tumorigenicity of Tsc2-deficient cells (Figure 4). Although COX2 is known to activate AKT [25, 26], COX2 overexpression had minimal effect on the activity of AKT in Tsc2-null cells (Supplementary Figure S3). Thus, in addition to inactivating AKT, mTORC1 can ameliorate TSC tumors through suppression of COX2 expression. We postulated that the decrease in COX2 in parallel with the attenuated AKT activity negatively regulates tumor growth in TSC. These findings could shed light on the mechanism underlying the benign tumor formation seen in multiple organs of TSC patients. In addition, our observation that loss of TSC2 suppressed COX2 expression through activation of mTORC1 may explain why COX2 is downregulated in renal carcinomas in the TSC2 gene mutant Eker rat [27].

We also demonstrated that mTORC1 inhibits COX2 expression in Tsc2-deficient cells through activation of STAT3, which is a known downstream effector of TSC2/mTORC1 signaling [28]. We previously reported that mTORC1-triggered tumorigenesis can be attributed, at least in part, to STAT3-mediated inhibition of cell differentiation and upregulation of glycolysis [22, 23]. Although STAT3 is widely considered to be an oncogene, emerging evidence suggests STAT3 may also suppress tumor growth. For example, it was recently reported that STAT3 negatively regulates thyroid tumorigenesis [29]. In the present study, we found that STAT3 inhibits COX2 transcription in response to dysregulated TSC1/mTORC1 signaling. The resultant downregulation of COX2 diminished the tumorigenicity of Tsc2-deficient cells. It is thus likely that STAT3 has a dual role in the development of TSC tumors. Caution should therefore be used when treating TSC and related cancers by targeting STAT3.

Given that aberrant activation of mTORC1 likely triggers TSC tumor formation [1, 4], it has been suggested that rapamycin and its analogues could potentially be effective for the treatment of TSC and related cancers [8, 30, 31]. Here, we found that rapamycin upregulated COX2 expression (Figure 2). We also observed that overexpression of COX2 accelerated cell proliferation and tumoral growth of Tsc2-null cells (Figure 4). It is therefore reasonable to assume that combined administration of COX2 inhibitors with rapamycin would help to eliminate the unwanted effects of the upregulated COX2 and improve the antitumor effect of rapamycin in the treatment of TSC. Consistent with that idea, after combined administration of rapamycin and celecoxib, we observed a strong inhibitory effect on the proliferation of Tsc2-null cells (Figure 7). This suggests co-administration of COX2 inhibitors and rapamycin may be an effective novel strategy for the treatment of TSC and related tumors.

IL-6 is a cytokine involved in many cell processes, including immune responses, cell proliferation, and tumorigenesis [32, 33]. Although the functional role and regulatory mechanism of IL-6 in TSC are less well characterized, several studies have reported that rapamycin promotes IL-6 production in different cell types. For example, Weichhart and collaborators showed that rapamycin enhances lipopolysaccharide-induced IL-6 production in human peripheral blood mononuclear cells [34]. In addition, Su et al. found that rapamycin treatment led to upregulated production of IL-6 in human macrophages [35], while Chen and colleagues reported that rapamycin promotes IL-6 secretion in rat Kupffer cells [36]. Consistent with these studies, we found that rapamycin increased the expression and secretion of IL-6 in mouse and rat Tsc2-deficient cells (Figure 6). Moreover, we identified COX2 as a downstream target of mTORC1 that promotes expression of IL-6 (Figure 5). It may thus be a common phenomenon across species that rapamycin stimulates COX2/IL-6 signaling through inhibition of mTORC1. In addition, Our finding that IL-6 is a downstream target of COX2 may also explain earlier observations that sphingosine-1-phosphate markedly induces COX2 expression and IL-6 secretion in human tracheal smooth muscle cells [37], and that administration of celecoxib significantly reduces serum IL-6 levels in patients with major depressive disorder [38].

In summary, this study demonstrated that activation of an mTORC1/STAT3 signaling cascade caused by a deficiency in TSC2 leads to downregulation of COX2 (Figure 7G). The decrease in COX2 blocks IL-6 production, thereby inhibiting Tsc2-deficient cell proliferation (Figure 7G). These findings increase our understanding of benign tumor formation in TSC and of the novel network regulating COX2 transcription. They also suggest a new strategy for treating TSC using a combination of rapamycin with COX2 inhibitors.

## MATERIALS AND METHODS

### Reagents, plasmids, and antibodies

Rapamycin, S3I-201, and celecoxib were obtained from Selleck Chemicals (Houston, TX, USA). Lipofectamine 2000 and NuPAGE 4-12 % Bis-Tris gel were purchased from Life Technologies (Carlsbad, CA, USA). pLVX-IRES-Puro vector was from Clontech (Mountain View, CA, USA). Packaging vectors (pVSVG, pREV, and pMDL) and pGL3-Basic vector have been described previously [23, 39]. pBabe-puro and pBabe-puro-STAT3C (a constitutively activated STAT3, STAT3C) were kindly provided by Dr. Yu Zhang (Cancer Institute & Hospital, Chinese Academy of Medical Sciences). Antibodies specific to phospho-S6 (Ser235/236), Raptor, Rictor, COX2, STAT3, phospho-STAT3 (Tyr705), AKT1, and phospho-AKT (Ser473) were obtained from Cell Signaling Technology (Danvers, MA, USA). TSC2 and β-actin antibodies were obtained from Santa Cruz Technology (Santa Cruz, CA, USA).

### Cell lines and cell cultures

Mouse embryonic fibroblasts (MEFs) and rat uterine leiomyoma-derived Tsc2-null ELT3 cells were described previously [39, 40]. HEK 293T cells were obtained from the ATCC (Manassas, VA, USA). All cells were cultured in DMEM supplemented with 10% fetal bovine serum at 37°C in a humidified incubator containing 5% CO_2_.

### Western blot analysis

The kidney cystadenoma tissues and paratumor tissues from C57BL/6 Tsc2+/− mice (17 months old) were extracted using RIPA buffer (Beyotime Biotechnology, Haimen, China). Western blot analysis of protein expression was performed as described previously [40]. In brief, whole cell or tissue lysates were normalized to the protein concentration using a Bradford assay (Bio-Rad, Hercules, CA, USA), separated on NuPAGE 4-12 % Bis-Tris gels (Life Technologies), transferred to PVDF membranes (Millipore, Billerica, MA, USA), and analyzed using the indicated antibodies. Immunoreactive protein bands were visualized using Pierce^TM^ ECL Western Blotting Substrate (Thermo Scientific, Waltham, MA, USA) and exposure to X-ray film.

### Quantitative real-time PCR (qRT-PCR)

Total RNA was isolated from cells using TRIzol reagent (Life Technologies) according to the manufacturer's instructions. First-strand cDNA was synthesized using a RevertAid™ First Stand cDNA Synthesis Kit (Fermentas, Waltham, MA, USA) according to the protocol provided by the manufacturer. Transcripts were detected using qRT-PCR carried out with SYBR Premix Ex Taq^TM^ II (TaKaRa, Shiga, Japan) according to the manufacturer's protocol in a StepOnePlus™ Real-Time PCR System (ABI, Foster City, CA, USA). The primer sequences used are listed in Supplementary Table S3.

### RNA interference

Cells were seeded into 12-well plates and transfected with siRNAs using Lipofectamine 2000 according to the protocol provided by the manufacturer. All siRNA oligonucleotides were synthesized by GenePharma (Shanghai, China). The siRNA target sequences are listed in Supplementary Table S4.

### Microarray analysis

Total RNA was isolated from approximately 1×10^7^ cells using TRIzol reagent (Life Technologies). The RNA quantification, microarray analysis, data processing and statistical analysis were performed by the Shanghai KangChen Bio-tech Company (Shanghai, China). The microarray experiments were performed using an Agilent Whole Mouse Gene Expression Microarray (4×44 K) or Agilent Whole Rat Gene Expression Microarray (4×44 K).

### Reporter constructs and luciferase reporter assay

A 1115-bp fragment of the rat COX2 promoter (−994/+121) was amplified by PCR using rat genomic DNA extracted from ELT3 cells and then cloned into the *Kpn* I/*Xho* III sites of the luciferase reporter plasmid pGL3-Basic (Promega, Madison, WI, USA). The primer sequences were 5′-CGGGGTACCCCCGGGCCAACACCA-3′ (forward) and 5′-CCGCTCGAGGCAGCAGTTGTGGCAGC-3′ (reverse). The potential STAT3-binding site on the promoter of the rat COX2 gene was mutated using Q5 Hot Start High-Fidelity DNA Polymerase (New England Biolabs, Ipswich, MA, USA). The primer sequences were 5′-AGTTATCGGCGCAAAAGTATTATCT-3′ (forward) and 5′-GGCCTTATTCTCGCTAACCTTAAAA-3′ (reverse). For Luciferase reporter assays, cells were cultured in triplicate to 80% confluence in 24-well plates and co-transfected with the promoter constructs (200 ng) and the internal control plasmid pRL-TK (20 ng). Luciferase activity was detected using a Dual-Luciferase Reporter Assay System (Promega).

### Recombinant plasmid construction, lentivirus production, and transduction

Full-length cDNAs encoding mouse COX2, rat COX2, and mouse IL-6 were acquired by PCR amplification from Tsc2+/+ MEFs or ELT3 cell cDNA pools and cloned into the pLVX-IRES-Puro vector. The recombinant plasmids and empty control vector were named as LV-COX2, LV-IL-6, and LV, respectively. The primers are listed in Supplementary Table S5.

GV248 lentiviral shRNA expression vectors targeting mouse COX2 and mouse STAT3, and the control scrambled shRNA (shSc) were obtained from Genechem (Shanghai, China). The target sequences were as follows: shCOX2^−1^, 5′-TACCCGGACTGGATTCTAT-3′; shCOX2^−2^, 5′-GCCATGGAGTGGACTTAAA-3′; shSTAT3, 5′-CTGG ATAACTTCATTAGCA-3′; shSc, 5′-AATCGCATAGCGT ATGCCG-3′.

HEK 293T cells were transfected with a recombinant vector or the corresponding control vector together with packaging plasmids (pVSVG, pREV, and pMDL). Culture supernatants were collected after 48 h of transfection and then used to infect target cells as described previously [39].

### Cell proliferation and cell viability assay

Cell proliferation was measured using MTT assays as described previously [23]. In brief, cells were plated into 96-well plates (0.5×10^3^-2.0×10^3^ per well) in triplicate, after which proliferation was monitored for up to 4 days in accordance with the manufacturer's instructions.

For the cell viability assays, 1.0×10^3^ cells were plated into in 96-well plates and then treated for 48 h with or without the indicated drugs. Cell viability was measured using MTT assays as described previously [41]. Cell viabilities were evaluated as relative values compared with untreated controls.

### Colony formation assay

Cells were dissociated into a single-cell suspension and then seeded into 10 cm cell culture dishes at a density of 50-100 cells/ml. The cells were cultured for about two weeks in DMEM containing 10% FBS with or without drugs, as indicated. After fixation, the cells were stained with 0.1% crystal violet (1 mg/ml), and colonies with more than 50 cells were counted.

### Tumorigenicity in nude mice

Tumorigenicity was assessed using immunodeficient BALB/c nude mice (5 weeks, 16-18 g) as described previously [23]. The mice were purchased from Vital River Laboratory Animal Technology (Beijing, China). For tumorigenicity assays, 4×10^6^ cells in 0.2 ml of DMEM were inoculated subcutaneously into the right anterior armpit of mice, and tumor growth was then monitored. The mice were euthanized and imaged on day 60 after inoculation, after which the tumors were excised and weighed. All animals were maintained and used in strict accordance with the guidelines of the Animal Center of Anhui Medical University, and all animal experimental procedures were approved by the Experimental Animal Ethical Committee of Anhui Medical University.

### Immunohistochemistry (IHC) analysis

Paraffin-embedded tissue blocks were cut into 4-μm slides. The histological sections were stained with hematoxylin-eosin (H&E) or immunostained with rabbit monoclonal antibodies against COX2 (Cell Signaling) or Ki-67 (Abcam, Cambridge, MA, USA) according to the manufacturer′s protocols.

### Enzyme-linked immunosorbent assay (ELISA)

Levels of IL-6 secretion were assessed using Valukine^TM^ IL-6 ELISA kits (R&D Systems, Minneapolis, MN, USA) following the manufacturer′s instructions. In brief, cells were seeded in 12-well plates in triplicate. After incubation for 12 h, the culture medium was replaced with 1 ml of serum-free medium with or without drugs, as indicated, and the incubation was continued for an additional 36 h. Cell culture supernatants were then harvested, and the IL-6 levels in the supernatants were measured using mouse or rat IL-6 ELISA kits (R&D Systems).

### Statistical analysis

Statistical tests were performed using GraphPad Prism 5 software. Differences between groups were analyzed using 2-tailed Student's t tests. Values of *P* < 0.05 were considered significant.

## SUPPLEMENTARY FIGURES AND TABLES


