# Supplementary material for: mTORC1-mediated downregulation of COX2 restrains tumor growth caused by TSC2 deficiency
Source: Oncotarget. 2016 Apr 7;7(19):28435–47. doi: 10.18632/oncotarget.8633 (PMC5053737; doi:10.18632/oncotarget.8633)
Supplement: Supplementary file 1 [file oncotarget-07-28435-s001.pdf]

SUPPLEMENTARY FIGURES AND TABLES

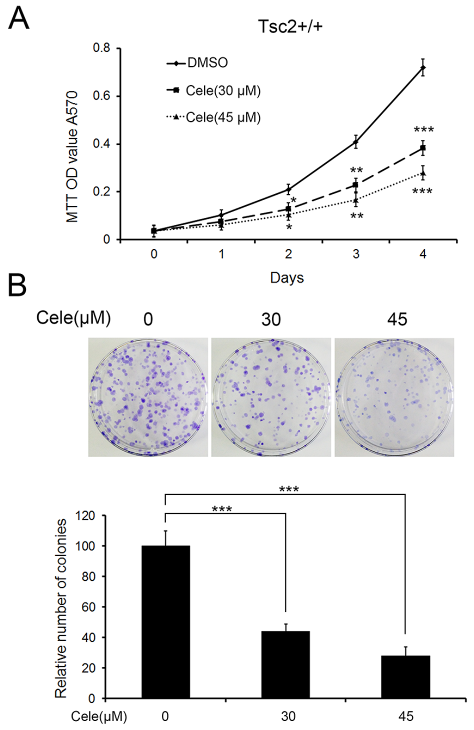

Supplementary Figure S1: Celecoxib treatment significantly inhibits Tsc2+/+ MEFs proliferation. Tsc2+/+ MEFs were treated with or without the indicated concentration of celecoxib (Cele). **A.** Proliferation of the indicated cells was examined using MTT assays. **B.** Representative images (upper panel) and quantifications (lower panel) of crystal violet-stained colonies formed by the indicated cells. Error bars indicate mean ± SD of triplicate samples. **P*<0.05; ***P*<0.01; ****P*<0.001.

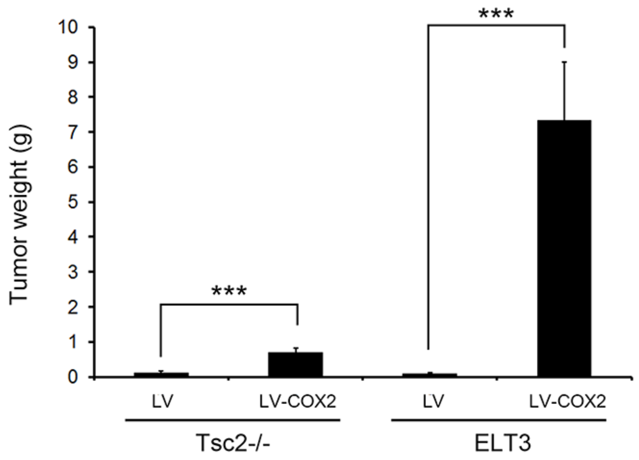

Supplementary Figure S2: Overexpression of COX2 enhanced the tumorigenicity of Tsc2-/- MEFs or ELT3 cells. Tsc2-/- MEFs or ELT3 cells infected with lentivirus harboring pLVX-IRES-Puro vector encoding COX2 (LV-COX2) or its empty control vector (LV) were subcutaneously injected into nude mice. The mice were sacrificed on day 60 after inoculation, after which the tumors were dissected and weighted. Error bars indicate mean ± SD. ****P*<0.001.

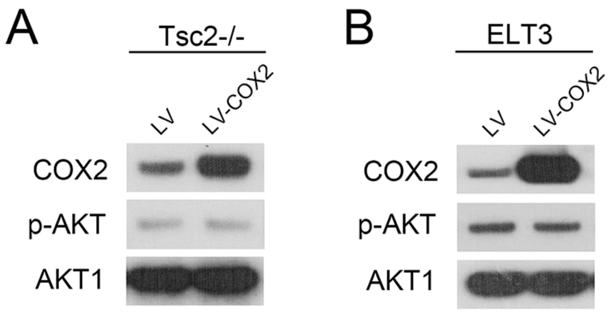

Supplementary Figure S3: Overexpression of COX2 has no effect on AKT activity in Tsc2-/- MEFs or ELT3 cells. Tsc2-/- MEFs (A) or ELT3 (B) cells were infected with lentivirus harboring pLVX-IRES-Puro vector encoding COX2 (LV-COX2) or its empty control vector (LV). The proteins were detected by immunoblotting with the indicated antibodies.

Supplementary Table S1: Knockdown of STAT3 led to upregulated expression of COX2 mRNA in Tsc2-/- MEFs.

Gene symbol	Fold-Change Tsc2-/- shSTAT3 vs Tsc2-/- shSc	Tsc2-/- shSTAT3 vs Tsc2-/- shSc (Description)
COX2	2.25	Up

The mRNA abundance of COX2 in Tsc2-/- shSc and Tsc2-/- shSTAT3 MEFs was measured by using Agilent Whole Mouse Gene Expression Microarray (4×44 K).

Supplementary Table S2: A list of the 41 commonly upregulated genes in COX2-overexpressing Tsc2-/- MEFs or ELT3 cells compared with their corresponding control cells.

Gene symbol	Fold-Change Tsc2-/- LV-COX2 vs Tsc2-/- LV	Fold-Change ELT3-LV-COX2 vs ELT3-LV
Atp10a	3.29	2.02
Atp2b4	3.22	2.94
Cdk14	3.20	2.69
Cftr	2.88	3.79
Chd2	2.73	2.22
Dll3	2.37	2.16
Dpp10	3.39	3.01
Etv2	4.37	5.31
Fam13c	11.26	3.89
Fam178b	3.58	2.12
Foxp2	2.32	3.05
Galnt15	2.86	3.97
Gnaz	5.91	2.26
Hist1h1a	2.23	4.56
**Il6**	**2.79**	**23.22**
Irg1	2.49	2.94

(Continued)

Gene symbol	Fold-Change Tsc2-/- LV-COX2 vs Tsc2-/- LV	Fold-Change ELT3-LV-COX2 vs ELT3-LV
Ism2	2.89	2.38
Klk14	93.36	5.67
Mab21l3	3.32	2.34
Mapk8	2.52	2.10
Masp1	2.13	2.60
Msh5	3.47	2.02
Nnmt	3.40	2.81
Nox4	2.90	8.23
Nr4a2	11.08	3.97
Pcdh9	3.43	9.73
Pde1a	3.98	5.16
Pde4b	2.58	2.25
Pet100	6.16	2.02
Pnma2	4.24	3.11
Pomc	2.21	3.06
Ptn	2.97	2.41
Ptprm	2.52	2.66
Rab9b	2.06	4.09
Rassf6	6.17	2.40
Rims2	2.94	2.49
Spint2	2.41	5.86
Svop	2.25	3.41
Trpm1	2.80	2.31
Zc3h6	3.14	3.42
Zfp536	2.01	2.19

Tsc2-/- MEFs or ELT3 cells were infected with lentivirus harboring pLVX-IRES-Puro vector encoding COX2 (LV-COX2) or its empty control vector (LV). The mRNA abundance was measured by using Agilent Whole Mouse Genome Oligo Microarray Kit (4 x 44K) or Agilent Whole Rat Genome Oligo Microarray (4 × 44 K).

Supplementary Table S3: The primer sequences used for qRT-PCR.

Target genes	Primer sequences (5′ to 3′)
mouse COX2 forward	TGAGCAACTATTCCAAACCAGC
mouse COX2 reverse	GCACGTAGTCTTCGATCACTATC
mouse IL-6 forward	TAGTCCTTCCTACCCCAATTTCC
mouse IL-6 reverse	TTGGTCCTTAGCCACTCCTTC
mouse β-actin forward	AGAGGGAAATCGTGCGTGAC
mouse β-actin reverse	CAATAGTGATGACCTGGCCGT
rat COX2 forward	CACGGACTTGCTCACTTTGT
rat COX2 reverse	GAACGCTTTGCGGTACTCAT
rat IL-6 forward	TGCCTATTGAAAATCTGCTCTGG
rat IL-6 reverse	ATTGGAAGTTGGGGTAGGAAGG
rat β-actin forward	AGAGGGAAATCGTGCGTGAC
rat β-actin reverse	GTGCTAGGAGCCAGGGCAGTA

Supplementary Table S4: The siRNA target sequences used in this study.

Target genes	Sequences (5′ to 3′)
mouse Raptor	GGACAACGGUCACAAGUAC
mouse Rictor	GCCCUACAGCCUUCAUUUA
mouse IL-6^{-1}	AGUCGGAGGCUUAAUUACA
mouse IL-6^{-2}	CAGGAAAUUUGCCUAUUGA
mouse IL-6^{-3}	UAAGGACCAAGACCAUCCA
rat Raptor	AUUACAGCAAGAAUGAAGG
rat Rictor	AUAGACCUAACUGAGGAGG
rat STAT3	GCAGAGUUCAAGCACCUGA
rat IL-6^{-1}	CCACAACAGACCAGUAUAU
rat IL-6^{-2}	GAGGCUUAAUUACAUAUGU
rat IL-6^{-3}	CUGGAUAUAACCAGGAAAU
negative control	UUCUCCGAACGUGUCACGU

Supplementary Table S5: The primer sequences used for plasmid construction.

Name	Sequences (5′ to 3′)
mouse-IL-6-*EcoR* I-forward	CCGGAATTCATGAAGTTCCTCTCTGC
mouse-IL-6-*BamH* I-reverse	CGCGGATCCTTAGCCACTCCTTCTGT
rat-COX2-*Spe* I-forward	GACTAGTATGCTCTTCCGAGCTGTGCTGCT
rat-COX2-*Not* I-reverse	ATAGTTTAGCGGCCGCTTACAGCTCAGTTG
mouse-COX2-*Spe* I-forward	GGACTAGTATGCTCTTCCGAGCTGTGCT
mouse-COX2-*BamH* I-reverse	CGCGGATCCTTACAGCTCAGTTGAAC
